# Heterogeneity in the effect of mid-childhood height and weight gain on human capital at age 14-15 years: Evidence from Ethiopia, India, Peru, and Vietnam

**DOI:** 10.1371/journal.pone.0212783

**Published:** 2019-02-22

**Authors:** Kaushalendra Kumar, Santosh Kumar, Ashish Singh, Faujdar Ram, Abhishek Singh

**Affiliations:** 1 Department of Public Health & Mortality Studies, International Institute for Population Sciences, Mumbai, Maharashtra, India; 2 Department of Economics and International Business, College of Business Administration, Sam Houston State University, Huntsville, Texas, United States of America; 3 Shailesh J. Mehta School of Management, Indian Institute of Technology Bombay, Mumbai, Maharashtra, India; Centre Hospitalier Universitaire Vaudois, FRANCE

## Abstract

**Background:**

Under-nutrition in early childhood has harmful impacts on human capital formation in children, with implications for educational, adult health, and labor market outcomes. We investigate the association of linear growth and weight gain in mid-childhood with years of schooling, the Peabody Picture Vocabulary Test score, and math test score during the adolescent age of 14–15 years.

**Methods:**

Data were derived from the Young Lives study conducted in four low- and middle-income countries (Ethiopia, India, Peru, and Vietnam). The data had detailed information on the children anthropometry and characteristics of the child, household, and community. Multivariate regression analysis, adjusted for the confounding variables, was used to investigate the association between mid-childhood health, measured by conditional linear growth and relative weight gain, and human capital outcomes in adolescent age.

**Results:**

After controlling for several confounders, one cm increase in conditional linear growth increased years of schooling by 0.034 years and the Peabody Picture Vocabulary Test score and math test score by 0.474 and 0.083 points respectively. Relative weight gain was negatively associated with years of schooling and math test score. There is no evidence of heterogeneous effects by rural, gender, and household wealth. In the quantile regression analyses, the association between conditional linear growth and outcomes is stronger at the lower level of years of schooling and the Peabody Picture Vocabulary Test score.

**Conclusion:**

Our study highlights that mid-childhood nutritional intervention targeted for students at the lower level of education distribution can accelerate the rate of human capital accumulation in low- and middle-income countries.

## Introduction

Human capital accumulation is often considered as the end and means of economic development. Human capital such as education and health can directly contribute to the output growth through increased productivity and technological progress. While exploring the determinants of human capital formation, a number of studies have found that fetal and early childhood malnutrition adversely affect later educational attainment, cognitive and non-cognitive skills, mid-childhood and adult health, labor productivity, and hence economic growth [1–4]. The effect of childhood nutrition on adult wage is mediated not only through improved physical health but also through better cognition development and higher educational attainment [5, 6].

Despite substantial evidence on the association between under-nutrition in early-life and subsequent cognitive development, very little is known about the relative importance of different phases of growth in early childhood. The literature on catch-up growth is mixed and unsettled. Some studies show that cognitive development responds more to investments made in the first 2 years of life [7] because growth failure stagnates after the first 2 years of life. Analyzing data from 54 resource-poor countries in Africa and Southeast Asia, authors articulated the first 100 days or the first 2 years of life as the “critical period” and interventions after age 2 are unlikely to have any effects in reversing the initial damage to cognitive development [8]. Studies show that schooling and cognitive achievements are strongly associated with early-childhood growth (the first 2 years) than mid-childhood growth [9–11]. However, in recent years several studies have found that physical growth failure can be reversed after the age of 2 years confirming that young children have the potential to grow and recover from stunting and underweight manifested in the first 2 years of life [12, 13]. These studies show that substantial height catch-up occurs between 2 years and mid-childhood and again between mid-childhood to adult years.

Given the importance of early life conditions on later human capital development, it is important to understand growth trajectories among children and its impacts on human capital. In low- and middle-income countries, growth faltering is very common during the first two years of life [8]. Height-for-age (HAZ) and weight-for-age (WAZ) declines considerably in the first 2 years of life and either stagnates or recovers partially after 2 years, implies that the first 2 years of life is the critical window for nutritional investment for preventing malnutrition in mid-childhood and adolescence [12]. Childhood malnutrition and slower weight and height gains in the first few years of life are mostly due to inadequate postnatal nutrition, which is caused by poverty and limited access to resources and health infrastructures.

In addition to examining the direct effect of linear growth and weight gain on schooling and cognitive outcomes, it is important to separate out the effect of weight gain from linear growth on schooling. Understanding which phase in childhood growth is associated with human capital may help policymakers to decide the timing of interventions to reduce the adverse effect of childhood malnutrition. Linear growth or height gain and weight gain may have different impacts on human capital, and therefore, it is very important to disentangle the effect of linear growth from that of weight gain [14]. However, identifying the specific age when linear growth and weight gain can predict later life outcomes is complicated by the serial correlation between height and weight at different ages. In addition, height and weight are correlated with each other. Weight gain in the first two years of life is less consistently associated with later outcomes. Studies have found that faster weight gain without concomitant linear growth in early childhood increases the risk of obesity and insulin resistance in adulthood [15–17]. Contrary to this, studies have also shown that faster linear growth poses a health risk in later life [18, 19]. Hence, there is a need to separate the effect of linear growth from weight gain on educational attainment by conducting conditional growth analyses.

A limited number of studies have attempted to separate out the effect of height gain from weight gain [10, 11, 14]. Conditional linear growth at age 2 was associated with an increase in IQ (4.28 points), years of schooling (1.58 years), and monthly income at age 30 in Brazil [10]. In contrast, relative weight gain was not associated with these outcome variables in Brazil. In a pooled analysis of 5 cohort studies from low- and middle-income countries, another study reported that linear growth not weight gain in the first 2 years of life was associated positively with years of schooling [14]. Early weight gain is either neutral or protective but later weight gain (after 2 years of life) is detrimental for future formation of human capital [8]. In another cohort studies conducted in Brazil, Guatemala, India, and the Philippines, it was found that conditional weight gain during the first 2 years of life was positively associated with years of schooling but weight gain between 2 and 4 years had no association with years of schooling [11].

The objective of the present study is to explore the association between conditional linear growth (CLG) and relative weight gain (RWG) between mid-childhood (7–8 years) and adolescence (14–15 years) on educational outcomes in four low- and middle-income countries (Ethiopia, India, Peru, and Vietnam). We estimate the mean effects in an ordinary least square (OLS) framework, while heterogeneity in the effects is examined by estimating an interaction model as well as quantile regression (QR) model.

## Materials and methods

### Data sources

Our study uses data from the Young Lives Study (YLS) of four prospective mid-aged (7–8 years) child cohorts from Ethiopia (N = 1000), India (N = 1008), Peru (N = 714) and Vietnam (N = 1000) surveyed first in 2002 and followed up during adolescence (14–15 years) in 2009–10. YLS was conducted by the University of Oxford, UK in collaboration with local institutions from each country and follows a purposive sampling strategy [20]. In each of the 20 sentinel site, households with a child in the required age range were identified and, from these 150 households (100 for the younger cohort and up to 50 for the older cohort) were randomly selected; the exact procedures varied between sites because of topographical and administrative differences within and between countries and were documented [20].

The YLS data is publicly available and could be obtained from the YL website (https://www.younglives.org.uk/). The older cohort consists of 3722 children born in 1994–95 and interviewed in 2001–02 (round 1). Of this sample, 3622 were reinterviewed in 2009 (round 3), leaving the attrition rate at 2.2%. Household mobility was the most important reason for attrition. However, the previous study found very little evidence of attrition bias [21]. Our analytical sample is derived from second and third rounds of YL data and consists of 3622 children from the older cohort who were interviewed in 2006 (round 2) and 2009 (round 3). The older cohort was 7–8 years old in 2002 and 14–15 years old in 2009. However, due to incomplete or missing information on confounding variables, our study used ~3500 children for the years of schooling and math test score model and ~2800 children for the Peabody Picture Vocabulary Test (PPVT) score model. The outcome and confounding variables were from first (2002) and third round (2009) of YL data, and, conditional growth variables (CLG and RWG) were based on first as well as the third round of YL data.

**Fig 1:**
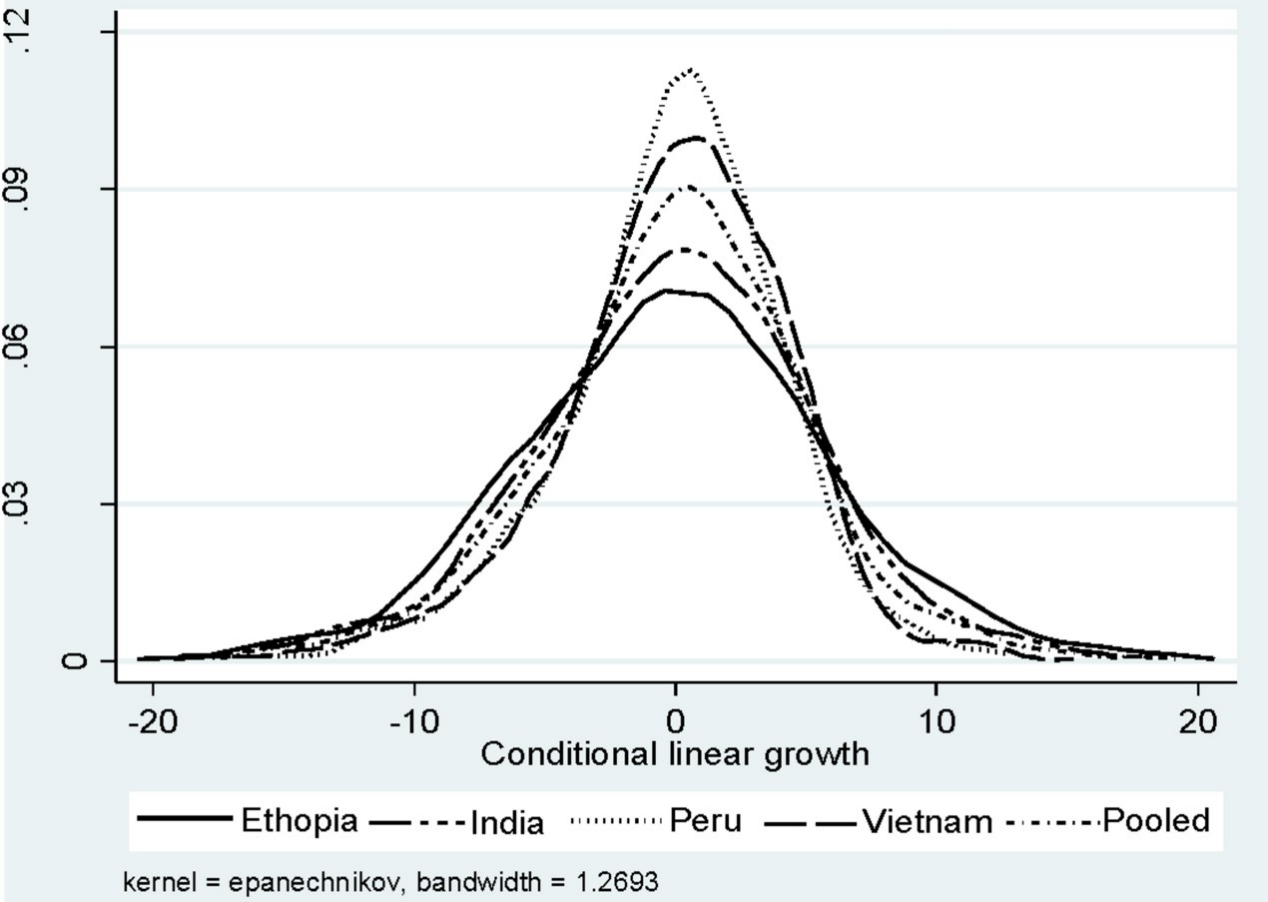
Kernel density of the conditional linear growth between 2002 & 2009, Ethiopia, India, Peru, Vietnam, and the pooled sample.

**Fig 2:**
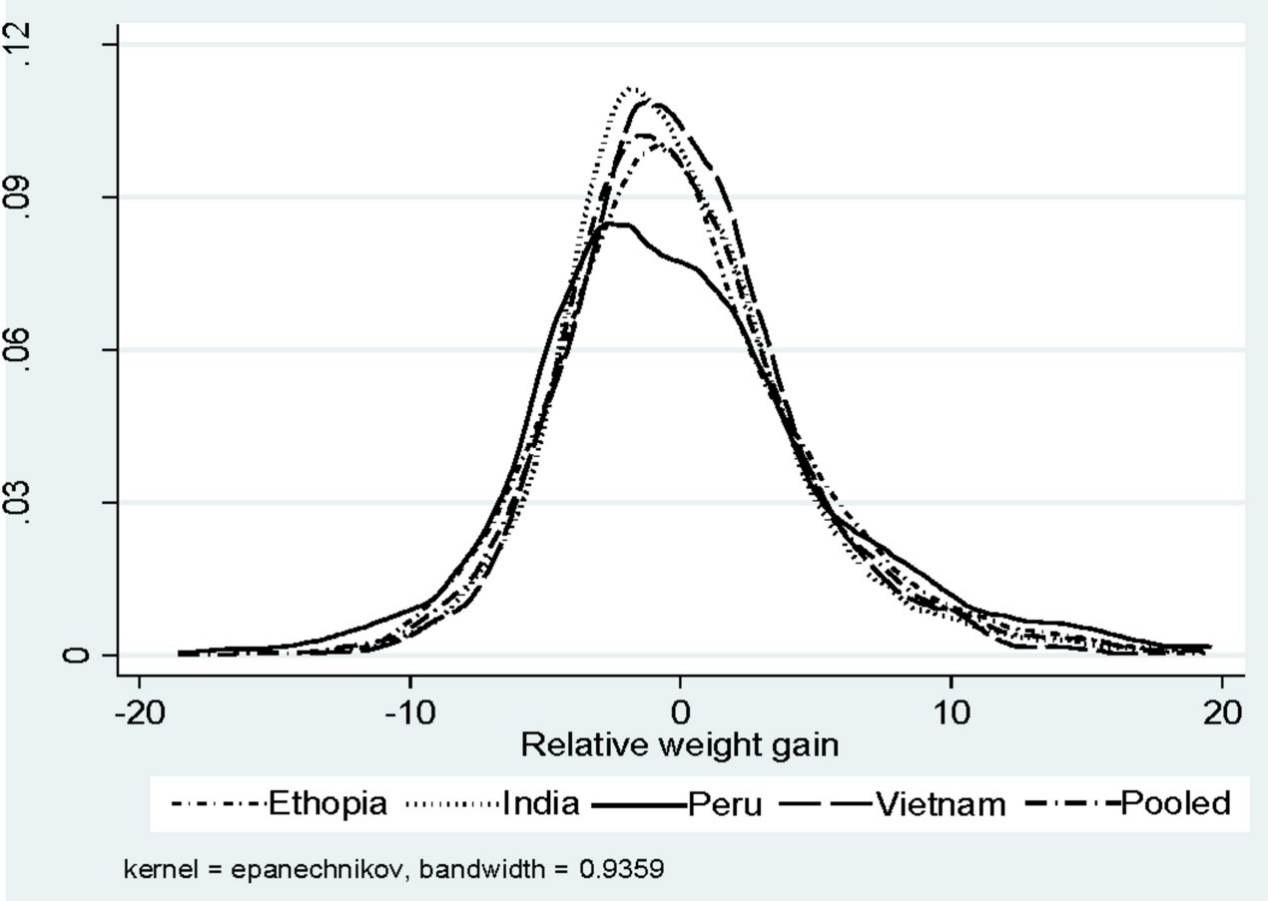
Kernel density of the relative weight gain between 2002 & 2009, Ethiopia, India, Peru, Vietnam, and the pooled sample.

Kernel Density graphs (Figs 1 and 2) show the estimated probability density function of conditional linear growth and relative weight gain in the four countries. It is clear from Figs 1 and 2 that the distributions of CLG and RWG are almost similar in all the four countries—Ethiopia, India, Peru, and Vietnam, however, levels are different. The data used is from the third round of the YL data, i.e. 2009. Therefore, our strategy to conduct the pooled analysis, i.e., combining the samples of the four countries are justified and the result may be interpreted as representative of the pooled data.

### Outcomes

The main outcome of interest in this study is the educational attainment of children during adolescence (age 14–15 years) in 2009. Educational attainment is measured by years of schooling, child’s receptive language skills (PPVT), and performance in standardized math test score, both of which were developed in YLS [22].

PPVT uses items that consist of a stimulus word and a set of pictures and is commonly used to represent child cognitive and intellectual ability in developing countries. The test requires respondents to select the pictures that best represent the meaning of a series of stimulus words read out by the surveyors. The test consists of 17 sets of 12 words each and the raw score test results can take possible values from 0 to 204. The PPVT of 125 questions was used in Peru, whereas PPVT of 204 questions was used in Ethiopia, India, and Vietnam. The PPVT was adapted and standardized by YL researchers in each country. The math test measures basic quantitative and number notions. It included 30 items on addition, subtraction, multiplication, division, data interpretation, problem-solving, and basic geometry. The total score of children in the math test was obtained from adding the correct responses and ranged from 0 to 30 [22]. The raw scores of PPVT and math test were used in the analysis.

### Conditional growth

The key explanatory variables are CLG and RWG during mid-childhood and adolescence. We calculated the CLG and RWG separately for males and females. The CLG of an adolescent is the expected adolescent height adjusted to their mid-childhood height and weight. Similarly, RWG is the expected adolescent weight accounting for the present height and mid-childhood weight and height. Empirically, CLG is the residual term of the regression of height at age 15 years on height and weight at age 8 years and observed height at age 15. Similarly, RWG is the residual term of the regression of weight at age 15 years on height and weight at age 8 years and observed weight at age 15 years. In other words, these conditional growth variables, CLG and RWG, are the deviations of the adolescent’s actual height or weight from the predicted conditional height or weight. Studies found that an individual attains the maximum physiological growth between mid-childhood (age 6–8 years) and adolescent (age 14–16 years) [23, 24]. Hence, our study captures the highest and optimal growth trajectory in the life course of educational attainment.

### Confounding variables

We also control for social-demographic (age, birth order, and gender of the indexed child; age of the mother at the birth of the indexed child; education of mother and father; caste/social group; religion/ethnicity; height of mother; morbidity at the time of early childhood; morbidity in last three years; number of times meal taken on the previous day of the survey) and household wealth and place of residence (rural versus urban) as these factors are likely to intermediate the effect of health on human capital accumulation [6]. Kumar (2016) shows that birth order is an important predictor of schooling outcomes, therefore, we include birth order in all models [25].

### Statistical analysis

Early childhood health status of an individual has a cumulative effect on the adolescent’s health and cognition development. Children with poor health experience low progress in height and low weight gain in due course of age advancement. Assume education is a functions of CLG or RWG between mid-childhood and adolescence:

Edu=f(CLGorRWG,C,P,S,η,ε) (1)

where, educational outcome measures *(Edu)* are the functions of CLG or RWG, individual level demographic characteristics (*C*), parental socio-demographic and economic characteristics (*P*), household socio-economic characteristics (*S*), sentinel or village/ward level fixed effect (*η*), and an error term (*ε*).

Empirically, we estimate the following models. First, we estimate the CLG and RWG model.

$$Height_{15}=\alpha_{1}+\beta_{1}Height_{8}+\beta_{2}Weight_{8}+\epsilon_{1}$$ (2)

$$CLG_{15}=Height_{15}-\hat{Height_{15}}$$ (3)

Similarly, RWG is estimated as follows:

$$Weight_{15}=\alpha_{2}+\beta_{3}Height_{15}+\beta_{4}Weight_{8}+\beta_{5}Height_{8}+\epsilon_{2}$$ (4)

$$RWG_{15}=Weight_{15}-\hat{Weight_{15}}$$ (5)

Where *Height*_8_ *and Weight*_8_ are height and weight during the mid-childhood period of age 7–8 years, whereas *Height*_15_ *and Weight*_15_ are height and weight during the adolescence period of age 14–15 years. *CLG*_15_ and *RWG*_15_ are conditional linear growth and relative weight gain at age 14–15 years, respectively. And, finally, the education equation can be estimated as the following Ordinary Least Square (OLS) model:

$$Edu_{15}=\alpha +\beta_{1}{\left(CLG\;or\;RWG\right)}_{15}+\beta_{2}C+\beta_{3}P+\beta_{4}S+\eta_{v}+\epsilon_{3}$$ (6)

*Edu*_15_ denotes years of schooling and test scores (PPVT and math test scores) at adolescence. The confounding variables (C, P, and S) are from the period when children are 7–8 years and 14–15 years old. We include *η*_*v*_, village fixed-effects, to control for fixed characteristics of villages. Any time-invariant differences between villages will be adjusted for by *η*_*v*_. Standard errors are clustered at the village level. In order to test whether the association between the conditional growth and outcomes differ by household characteristics, we estimate interaction models. We interact the conditional growth variables in Eq (6) by rural, the gender of the child, and the wealth level of the households.

A number of studies have shown that nutritional supplementation among the severely stunted children has a stronger effect on the recovery of their cognition development than the relatively healthy ones [26, 27]. This implies that CLG and RWG will have a larger effect on human capital at the lower level of the educational distribution relative to the higher level of educational distribution. Therefore, CLG and RWG elasticity of educational attainment depend on the distribution of education levels.

Therefore, we estimate the quantile regression (QR) model to capture the underlying heterogeneity in the relationship between CLG/RWG and educational outcomes. We estimate the relationship in Eq (6) at different quantiles of the conditional distribution of educational outcomes for *θ* ∈ {0.1, 0.25, 0.5, 0.75, 0.9}. This allows us to measure the effect at each of three quartiles and at the first and last decile. This helps us to gain a comprehensive view of how the relationship changes with the distribution of educational outcomes. It is important to note that that QR is not the same as mean regression applied to different subsets of the data ordered by the distribution of the dependent variables. The QR at *θ* = 0.1 is very different from estimating a mean regression where we condition on data in the low tail of the distribution. Since the cumulative distribution of the CLG and RWG is similar in all countries, we have carried out the analysis on the pooled data of all the four countries.

Furthermore, there may be a case that, some unobserved characteristics like genetics, maternal characteristics, household or community environment, public program etc. might have caused some children to record higher CLG and RWG than the others. Due to unobserved time-invariant heterogeneity, error term in Eq (6) may be correlated with the exposures variable. Hence, CLG and RWG may be endogenous. Nevertheless, panel structure of data and the fact that CLG and RWG, the two key explanatory variables are measured as the deviation of the observed value from the expected value should be able to address this concern. In addition, village fixed-effects will also address this concern. However, our study is unable to address the endogeneity concern if it is stemming from unobserved time-variant factors. Therefore, the causal interpretation of our estimates would be weak in the presence of time-variant unobserved factors.

## Results

We present the sample means of the outcome variables, key independent variables, and control variables in Table 1. In the pooled sample, 57.6% of the sampled children have completed secondary education and the average years of schooling is 6.8 years. The mean PPVT and math test-score are 136.4 and 11.0, respectively. Between mid-childhood to adolescent average height has increased from 118.3 cm to 154.4 cm, whereas weight has increased from 20.3 kg to 43.6 kg. On average, 52% of the sample has recorded positive CLG while 46% achieved positive RWG. The average age of children is approximately 179.8 months or 14.9 years in 2009. About 51% of the children are male and the mean birth order is 2.17. The percent of rural households is 65. Close to half of the parents had completed primary education.

**Table 1:** Descriptive statistics of educational outcome measures and exposure variables (N = 3604).

	Mean	Std. Dev.
Years of schooling 2002-Round1	2.44	1.20
Years of schooling 2009-Round3	6.76	2.94
PPVT score 2009-Round3	136.38	41.61
Math test score 2009-Round3	11.00	7.97
Positive conditional linear growth during 2002–2009	0.52	0.50
Positive relative weight gain during 2002–2009	0.46	0.50
Height (cm) 2002-Round1	118.30	6.35
Height (cm) 2009-Round3	154.35	8.23
Weight (kg) 2002-Round1	20.34	3.60
Weight (kg) 2009-Round3	43.56	8.70
Current age (months) 2002-Round1	95.28	3.65
Current age (months) 2009-Round3	179.82	3.97
Illness in the last three years 2002-Round1	0.14	0.35
Illness in the last three years 2009-Round3	0.25	0.43
Wealth index score 2002-Round1	0.38	0.22
Wealth index score 2009-Round3	0.51	0.21
# of times meal was taken on the previous day 2009-Round3	5.14	1.16
Male	0.51	0.50
Birth order	2.17	4.95
Mother’s height (cm)	152.85	8.63
Mother’s age at child’s birth (year)	24.73	6.41
Mother completed primary and above education	0.47	0.50
Father completed primary and above education	0.52	0.50
Rural	0.65	0.48

The correlation matrix presented in Table 2 shows the correlation between the explanatory variables- CLG, RWG, and outcome variables- years of schooling, the PPVT, and math test-score. CLG is positively associated with years of schooling (*ρ*: 0.11), PPVT score (*ρ*: 0.13), Math test-score (*ρ*: 0.10). Relative weight gain is positively correlated with the PPVT score (*ρ*: 0.03). Outcome variables are positively correlated among themselves, but a low value of correlation coefficient (≤ 0.54) implies that the three measures capture different dimensions of human capital.

**Table 2:** Correlation matrix.

	Conditional linear growth	Relative weight gain	Years of schooling	PPVT score	Math test score
Conditional linear growth	1.00				
Relative weight gain	0.00(1.00)	1.00			
Years of schooling	0.11(0.00)	-0.02(0.19)	1.00		
PPVT score	0.13(0.00)	0.03(0.09)	0.36 (0.00)	1.00	
Math test score	0.10(0.00)	0.00(1.00)	0.54 (0.00)	0.48 (0.00)	1.00

Note: Standard errors are in parenthesis.

Table 3 presents the results of the OLS regression of CLG on educational outcomes, adjusted for the confounding variables. The result shows that CLG has a significant positive association with all three measures of education. An increase in CLG by one cm increases years of schooling by 0.034 years, PPVT score by 0.474 points, and math test score by 0.083 points. All three coefficients are statistically significant at 1 percent level of significance. To put the CLG magnitude in perspective, 30 cm (12 inches) positive deviation in the height of the adolescent from the expected linear growth and weight gain would increase years of schooling by one year (30x0.034 = 1.02 years), and PPVT and math test score by 14.2 (30x0.474 = 14.22 points) and 2.5 points (30x0.083 = 2.49 points), respectively.

**Table 3:** Effect of conditional linear growth and relative weight gain on educational outcomes.

	Years of schooling	PPVT score	Math test score
**Panel A**			
Conditional linear growth (CLG)	0.034***	0.474***	0.083***
	(0.008)	(0.100)	(0.018)
Village fixed effect	Yes	Yes	Yes
R-squared	0.16	0.10	0.23
Observations	3,542	2,863	3,533
**Panel B**			
Relative weight gain (RWG)	-0.026**	0.026	-0.048*
	(0.009)	(0.107)	(0.020)
Village fixed effect	Yes	Yes	Yes
R-squared	0.16	0.09	0.23
Observations	3,542	2,863	3,533

*Note*: Standard errors clustered at the village level are in parenthesis. *Control variables*: age, sex, birth order, illness in the three years prior to survey years (2002 and 2009), mother’s height, mother’s age at birth of the index child, mother’s education, father’s education, education expenditure, wealth tercile 2002 & 2009 and place of residence.

* p < 0.05,

** p < 0.01,

*** p < 0.001.

The results on RWG demonstrate that RWG has a significant negative effect on years of schooling and math test score. On an average, one kg deviation in RWG results in a decline in years of schooling by 0.026 years and math test score by 0.048 points. The effect of RWG on PPVT score is positive but the estimates are statistically significant. The negative effect of RWG on years of schooling and math test score is consistent with previous findings that show that conditional weight gain is associated with poor health and higher risks of metabolic and cardiovascular disease and thereby adversely affect years of schooling and IQ [10].

The estimates for heterogeneous analyses, based on the interaction model, are reported in Table 4. The goal here is to explore if the association between CLG/RWG and educational outcomes vary by child or household’s characteristics, particularly by rural-urban residence, by gender of the child, and by household wealth. The estimates in Table 4 suggest that the effect of CLG on years of schooling is higher in rural areas compared to urban areas. The association between CLG and years of schooling is 0.03 years higher in rural areas compared with urban areas. Similarly, the interaction coefficient for PPVT score is positive meaning that condition linear growth would have a higher impact on PPVT score in rural areas than the urban areas, however, the estimate is statistically insignificant. There is no evidence that the effects vary by the gender of the child. The interaction coefficient (CLG*female) is always statistically insignificant and signs are not consistent. Furthermore, the association between CLG and outcomes do not vary by household wealth. Compared to the children in the top wealth group, children in the bottom wealth group are more likely to benefit from conditional growth but the estimates are not statistically significant. The estimates for relative weight gain further indicate that RWG association with all the three outcomes does not differ by rural, gender, and wealth of the household. None of these coefficients are statistically significant for RWG variable.

**Table 4:** Heterogeneous effects of conditional linear growth and relative weight gain on educational outcomes^@^.

	Conditional linear growth (CLG)			Relative weight gain (RWG)		
	Years of schooling	PPVT score	Math test score	Years of schooling	PPVT score	Math test score
CLG*rural	0.033*	0.264	-0.0007	-	-	-
	(0.016)	(0.206)	(0.037)	-	-	-
CLG*female	-0.001	0.149	-0.12	-	-	-
	(0.015)	(0.199)	(0.035)	-	-	-
CLG*poorest	0.022	0.074	0.034	-	-	-
	(0.015)	(0.201)	(0.035)	-	-	-
CLG*middle	-0.0009	-0.033	-0.045	-	-	-
	(0.016)	(0.208)	(0.036)	-	-	-
RWG*rural	-	-	-	-0.012	0.007	0.035
	-	-	-	(0.017)	(0.213)	(0.040)
RWG*female	-	-	-	0.011	-0.241	0.029
	-	-	-	(0.017)	(0.208)	(0.039)
RWG*poorest	-	-	-	-0.026	0.101	0.059
	-	-	-	(0.019)	(0.240)	(0.048)
RWG*middle	-	-	-	0.009	0.181	0.004
	-	-	-	(0.018)	(0.224)	(0.042)

^*@*^*Note*: Standard errors clustered at the village level are in parenthesis. Each coefficient is from a separate regression.

*Control variables*: age, sex, birth order, illness in the three years prior to survey years (2002 and 2009), mother’s height, mother’s age at birth of the index child, mother’s education, father’s education, education expenditure, wealth tercile 2002 & 2009 and place of residence.

* p < 0.05,

** p < 0.01,

*** p < 0.001.

Quantile regression coefficient of CLG on educational outcome measures for the pooled data is shown in Table 5. Results show that quantile estimates of CLG at the lower (25^th^) and middle (50^th^) quantile of years of schooling has a statistically significant effect on years of schooling. The effect size (0.029) at the 25^th^ quantile is approximately 50% larger than the effect size at the 50^th^ quantile (0.014). However, there is no effect at the higher quantiles reflecting a weaker association between CLG and years of schooling at a higher level of schooling. Effect of CLG on PPVT score increases from 0.497 at the 10^th^ quantile to 0.573 at the 25^th^quantile, thereafter quantile regression coefficient consistently declines up to 90^th^ quantile of PPVT score (0.188). Further, the estimated effects of CLG on math test score consistently increase from 0.060 to 0.093 between 10^th^ and 75^th^ quantiles. The estimated effects of CLG on math test score at the 75^th^ quantile are one and half times the magnitude of the estimated effect at the 10^th^ quantile.

**Table 5:** Quantile regression coefficients of CLG on educational outcomes^@^.

	*Quantile10*	*Quantile25*	*Median*	*Quantile75*	*Quantile90*
Years of schooling	0.008	0.029**	0.014*	0.000	0.000
	(0.01)	(0.01)	(0.006)	(0.005)	(0.002)
PPVT score	0.497*	0.573**	0.284**	0.233*	0.188*
	(0.201)	(0.142)	(0.105)	(0.098)	(0.087)
Maths Test score	0.060**	0.067**	0.083**	0.093**	0.062*
	(0.018)	(0.017)	(0.022)	(0.026)	(0.027)

^*@*^*Note*: Standard errors clustered at the village level are in parenthesis. Each coefficient is from a separate regression.

*Control variables*: age, sex, birth order, illness in the three years prior to survey years (2002 and 2009), mother’s height, mother’s age at birth of the index child, mother’s education, father’s education, education expenditure, wealth tercile 2002 & 2009 and place of residence.

* p < 0.05,

** p < 0.01,

*** p < 0.001.

Table 6 shows the quantile regression coefficient of RWG on educational outcome measures. Results show that there is negative RWG gradient in years of schooling and math test score. The negative effect of RWG on years of schooling declines between 10^th^ quantile (*β* -0.028) and median (*β*-0.009). None of the quantile regression coefficients for PPVT score are statistically significant at any quantiles. The negative effect of RWG on the math test score is -0.040 at the 25^th^ quantile of the math test score distribution. Overall, there is some evidence of stronger association at the lower distribution of outcome, particularly for CLG.

**Table 6:** Quantile regression coefficients of RWG on educational outcomes^@^.

	*Quantile10*	*Quantile25*	*Median*	*Quantile75*	*Quantile90*
Years of schooling	-0.028*	-0.020*	-0.009	0.000	0.000
	(0.011)	(0.005)	(0.001)	(0.002)	(0.002)
PPVT score	0.145	0.009	0.051	-0.110	-0.039
	(0.142)	(0.105)	(0.081)	(0.069)	(0.070)
Maths Test score	-0.010	-0.040*	-0.032	-0.022	-0.025
	(0.023)	(0.02)	(0.029)	(0.027)	(0.030)

^*@*^*Note*: Standard errors clustered at the village level are in parenthesis. Each coefficient is from a separate regression.

*Control variables*: age, sex, birth order, illness in the three years prior to survey years (2002 and 2009), mother’s height, mother’s age at birth of the index child, mother’s education, father’s education, education expenditure, wealth tercile 2002 & 2009 and place of residence.

* p < 0.05,

** p < 0.01,

*** p < 0.001.

**Fig 3:**
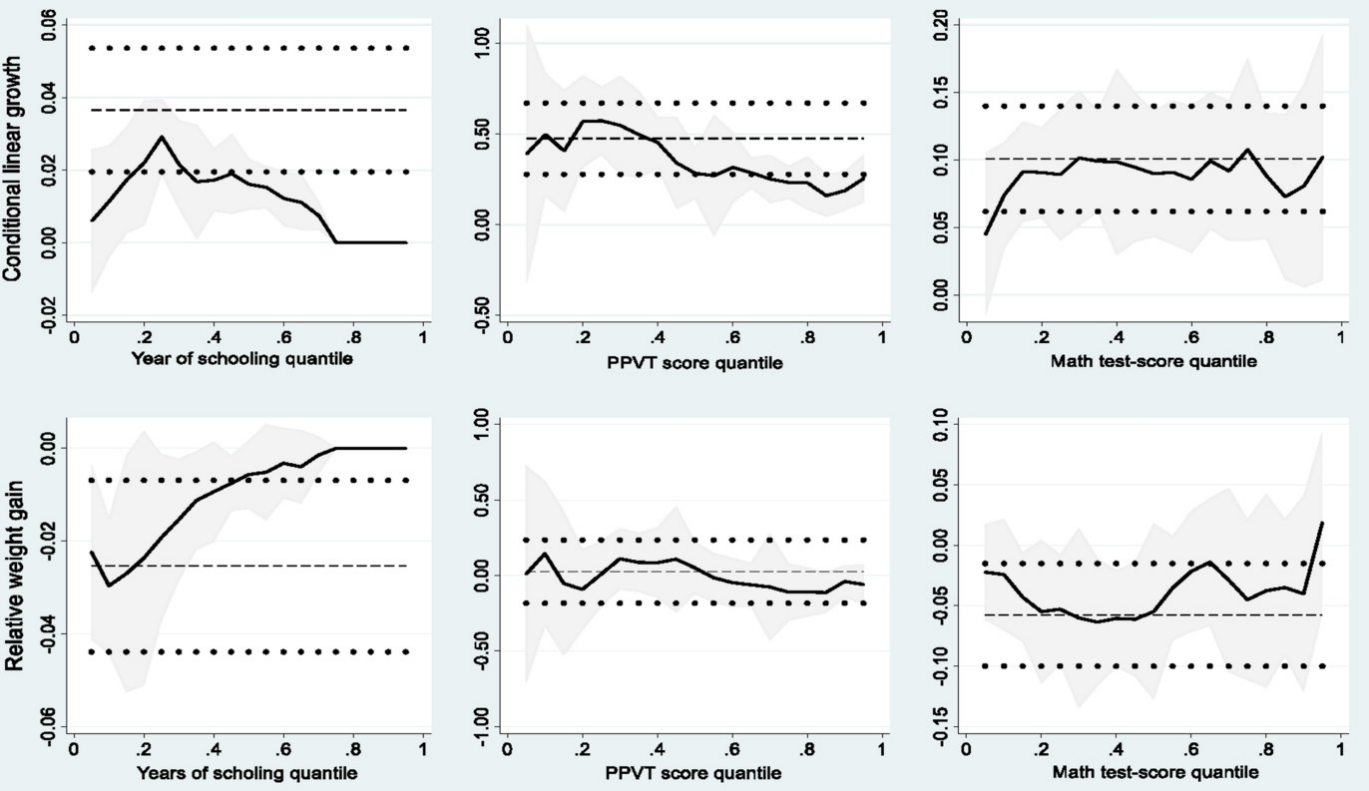
OLS and quantile regressions of CLG and RWG on educational outcomes.

Fig 3 which presents the OLS and quantile regression coefficients of CLG and RWG on educational outcome measures, respectively clearly show that for many quantiles the quantile regression coefficients lie outside the 95% confidence interval of the OLS coefficients. Therefore, the effect of CLG and RWG on educational outcomes clearly varies with the distribution of outcome variables. This justifies the use of quantile regression to capture the heterogeneity in the effects of CLG/RWG at different distributions of outcome variables.

## Discussion

The present study is among the handful of studies that investigate the effects of CLG and RWG on educational attainment in four low- and middle-income countries (Ethiopia, India, Peru, and Vietnam). This study adds to the scant literature on the effect of conditional linear growth and relative weight gain on schooling achievement and test scores. With the recent growing evidence on the dual burden of undernutrition and obesity in developing countries, it is important to separate the effect of linear growth on educational attainment from the effect of weight gain on educational attainment. Using quantile regression and interaction models, we examine the heterogeneous effect of CLG and RWG on years of schooling, PPVT score, and math test score. Our study extends previous works by focusing on heterogeneity in the effects by sub-groups and by different quantiles of distribution of the years of schooling, PPVT score and math test score. Our findings will provide empirical evidence for the targeted intervention among the severely disadvantaged section of the population.

The correlation coefficient between CLG and RWG is zero, which shows that we have disentangled the effect of linear growth from the effect of weight gain on educational attainment. The results show that conditional linear growth has a significant positive effect on years of schooling, PPVT, and math-test score. The results show that 30 cm (12 inches) positive deviation in the height of the adolescent from the expected height as per mid-childhood height and weight will result in one more years of schooling, 14.2 and 2.5 points higher value of PPVT and math test score, respectively. Consistent with the previous literature, we also find that relative weight gain is negatively associated with educational measures. On average, one-kilogram positive deviation in adolescent weight as per mid-childhood height, weight and current height result in a decline in 0.026 years of schooling and 0.048 points math-test score. This implies that one-kilogram increase in RWG is associated with an increase of 0.026 and 0.048 in years of schooling and math test score, respectively. Results from the quantile regression model show evidence of heterogeneous effects; the magnitude of effects varies by the distribution and level of the dependent variables. Generally, the positive effects of height on educational outcome measures are more pronounced at the lower quantiles of years of schooling, PPVT, and math test score. However, no consistent pattern emerges for the distributional impacts of weight on educational outcomes.

Furthermore, nutritional supplementation up to three years of age has the maximum effect on educational attainment in adulthood [14, 28, 29]. Similarly, Duc & Behrman, 2017 have shown that early childhood nutrition status has heterogeneous effects on mid-childhood health outcomes [9]. Though these above studies have established the effect of child health on educational attainment, none of them has investigated the differential impact of health improvement on varying levels of educational outcome measures. Our study complements and adds to the existing literature by taking into account the heterogeneous effect of CLG and RWG on educational attainment by estimating a quantile regression model.

Our study has a few limitations, including a lack of causal relationships and lack of information on birth weight and anthropometrics before age 8, and data pooling. First, conditional height and weight gain are likely to be endogenous as they are correlated with changes in household wealth or socio-economic status and with changes in access to health and educational infrastructures as well. It is possible that there may be observed (household income/wealth) or unobserved factors affecting both CLG/RWG and the educational outcomes. For example, any negative health shock that causes health and weight gain to slow may have negative impacts on school attendance and participation as well. In this study, we are unable to address the causality issue and unobserved heterogeneity, primarily due to the lack of suitable instrumental variable that could address the endogeneity in CLG/RWG. Second, previous studies have found that early life nutrition and birth outcomes are important determinants of height/weight gain and educational outcomes. The analytical model in this study does not control for the initial height, birthweight or any other indicator of early-life health stock. Third, the study currently analyzes data pooled from all the four young lives countries. It is likely that some of the variables are not comparable across countries because of the difference in the scale used. More importantly, it is important to test whether a similar association is observed across different countries. Therefore, to check the external validity of our findings, it would be interesting to analyze each country sample separately. However, it is likely that country-level sample may not have sufficient sample size to conduct these analyses, especially the quantile regression modeling.

As our study highlights that mid-childhood nutritional intervention targeted for malnourished children at a lower level of distribution, in rural areas, and among poor children might accomplish a higher level of educational outcome. Our analysis also indicates that the estimation of central tendencies (OLS and median regression) may be misleading and quantile regression estimation may provide more reliable information about the vulnerable target groups. Given that childhood malnutrition in low- and middle-income countries is highly correlated with socioeconomic characteristics, one of the key reforms needed is that the nutritional intervention should focus on children belonging to socio-economic deprived sections, especially in rural areas and children from poor households. Our results suggest that investment on these vulnerable groups would maximize the effect of early life health on educational outcome later in life.
